# Supplementary material for: Rapid single-cell physical phenotyping of mechanically dissociated tissue biopsies
Source: Nat Biomed Eng. 2023 Apr 6;7(11):1392–403. doi: 10.1038/s41551-023-01015-3 (PMC10651479; doi:10.1038/s41551-023-01015-3)
Supplement: Supplementary file 1 — Figures, tables and references. [file 41551_2023_1015_MOESM1_ESM.pdf]

nature biomedical engineering

Article

https://doi.org/10.1038/s41551-023-01015-3

Rapid single-cell physical phenotyping of mechanically dissociated tissue biopsies

In the format provided by the authors and unedited

CONTENTS

1. Supplementary Figures

Supplementary Fig. 1 | Correlation of physical phenotype parameters obtained using RT-FDC and histopathology scoring of mouse transfer colitis samples.

Supplementary Fig. 2 | Exclusion of small cells from the analysis of healthy vs tumour murine colon samples.

Supplementary Fig. 3 | Principal component feature importance for mouse tumours.

Supplementary Fig. 4 | Correlation of deformation and cell size in murine healthy and tumour samples.

Supplementary Fig. 5 | Principal component feature importance for human biopsy samples.

Supplementary Fig. 6 | Testing the performance of healthy vs tumour classification of frozen human colon samples.

Supplementary Fig. 7 | Testing the performance of healthy vs tumour classification on reduced data from frozen human colon samples.

Supplementary Fig. 8 | Testing of the performance of logistic regression on mixed samples consisting of different ratios of tumour and healthy lung biopsy samples.

2. Supplementary Tables

Supplementary Table 1 | Standard dissociation protocols used for each organ.

Supplementary Table 2 | Mouse organs with the corresponding tissue grinder protocol and microfluidic chip size used for the RT-FDC measurement.

Supplementary Table 3 | Parameters derived from cell images in real-time during an RT-FDC measurement.

Supplementary Table 4 | Antibodies and fluorescent probe solutions used with the corresponding dilutions.

Supplementary Table 5 | Pathological information and stromal content evaluation of frozen human colon biopsy samples.

Supplementary Table 6 | Pathological information and stromal content evaluation of frozen human colon biopsy samples.

Supplementary Table 7 | Histopathological evaluation of frozen human colon biopsy samples.

Supplementary Table 8 | Histopathological evaluation of fresh human colon biopsy samples.

Supplementary Table 9 | Pathological information and stromal content evaluation of fresh human lung biopsy samples.

Supplementary Table 10 | Histopathological evaluation of fresh human lung biopsy samples.

Supplementary Table 11 | Deformability Cytometry Open Repository (DCOR) dataset identifiers.

Supplementary Figures

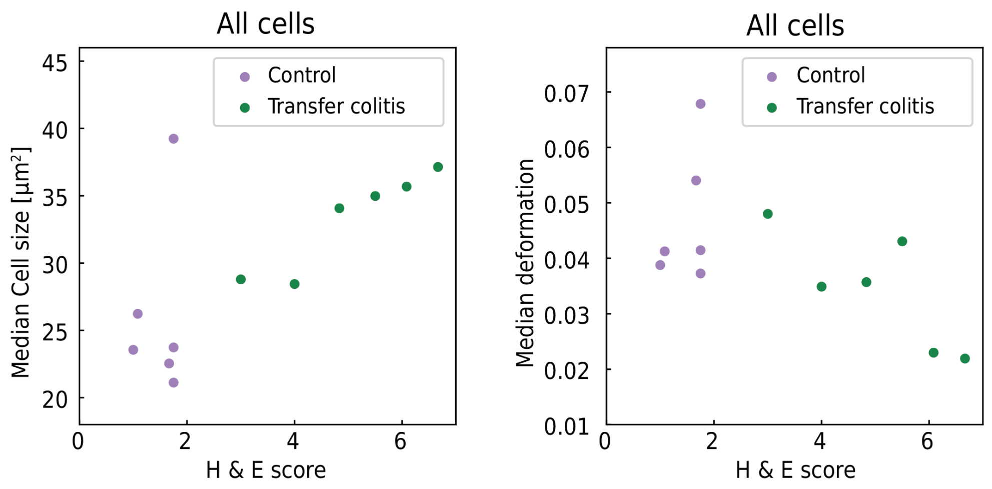

Supplementary Fig. 1 | Correlation of physical phenotype parameters obtained using RT-FDC and histopathology scoring of mouse transfer colitis samples. Plot of Haematoxylin & Eosin (H&E) scoring vs median cell size (left) and deformation (right) of all cells measured. Transfer colitis samples are shown in green, control (healthy) samples are shown in purple.

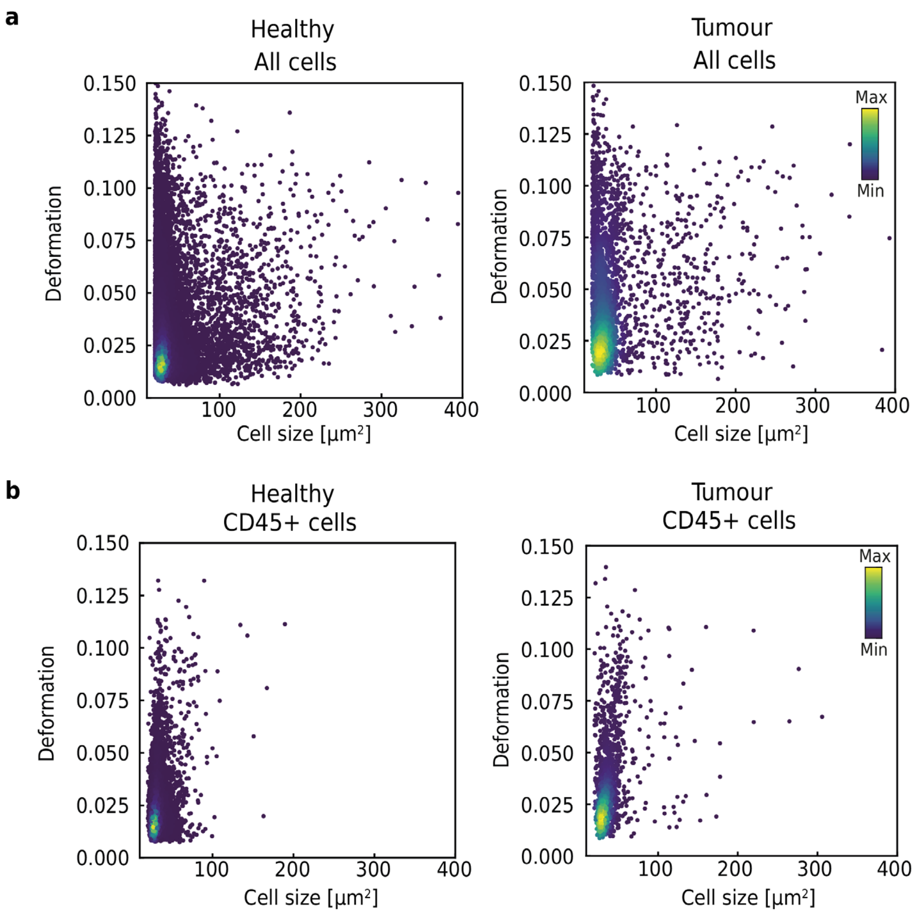

c

	Mean	STD
debris / all events < 60µm²	89.6%	10.5%
CD45+ cells / total cells < 60µm²	57.2%	19.0%

Supplementary Fig. 2 | Exclusion of small cells from the analysis of healthy vs tumour murine colon samples. a, Representative RT-FDC scatter plots of deformation vs cell size, showing cells isolated from tumour or healthy murine colon samples. **b,** Representative scatter plots of CD45 positive cells (a marker of leukocytes), showing that a high percentage of these cells are less than 60 µm^2 in size. These cells were excluded from the analysis. **c,** The percentage of debris in events less than 60 µm^2 in size and the percentage of CD45 positive cells, calculated as mean and standard deviation of all measured samples.

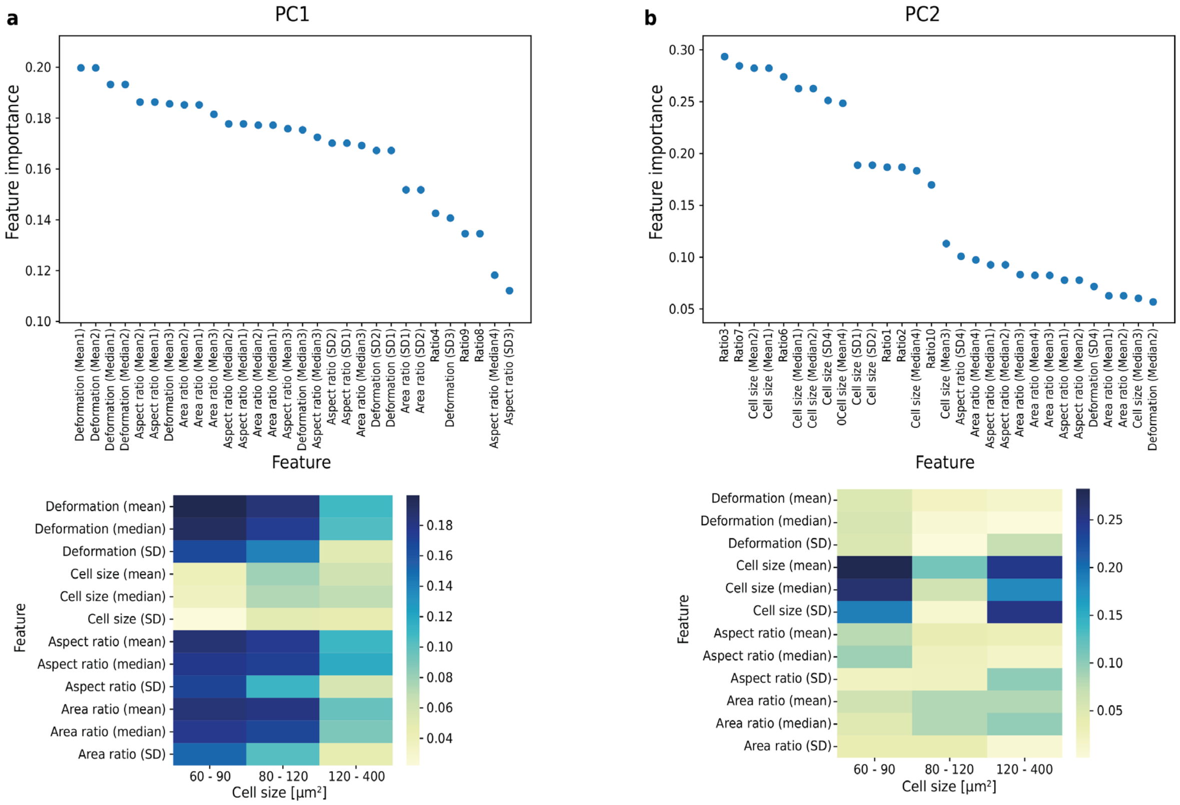

Supplementary Fig. 3 | Principal component feature importance for mouse tumours. PCA was performed on a total of 36 parameters; 12 parameters (y axis) for 3 cell size categories (x axis). **a,** Relative feature importance for PC1 and **b,** PC2. SD: standard deviation. The number at the end of each feature label describes which bin of cell sizes it corresponds to; 1: 60-90; 2: 80-120; 3: 120-400 μm^2.

a

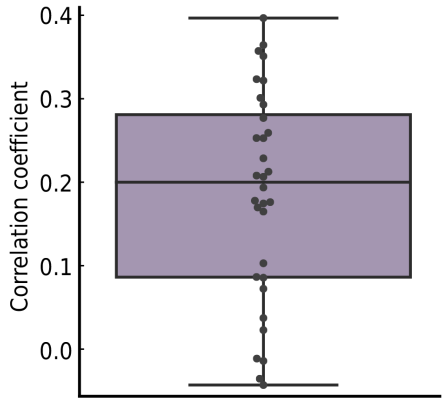

b

Correlation coefficients	Mean area and mean deformation	Median area and median deformation
Cells 60 - 90 µm^2	0.078	0.084
Cells 80 - 120 µm^2	0.064	0.174
Cells 120 - 400 µm^2	0.001	-0.008

Supplementary Fig. 4 | Correlation of deformation and cell size in murine healthy and tumour samples. a, Pearson's correlation coefficients for deformation and area; each point corresponds to one murine sample and b, table showing Pearson's correlation coefficients for the statistical means and medians of area and deformation, divided into the three cell size categories used for the principal component analysis. The box plot extends from the 25th to the 75th percentile with a line at the median; whiskers span 1.5x the interquartile range.

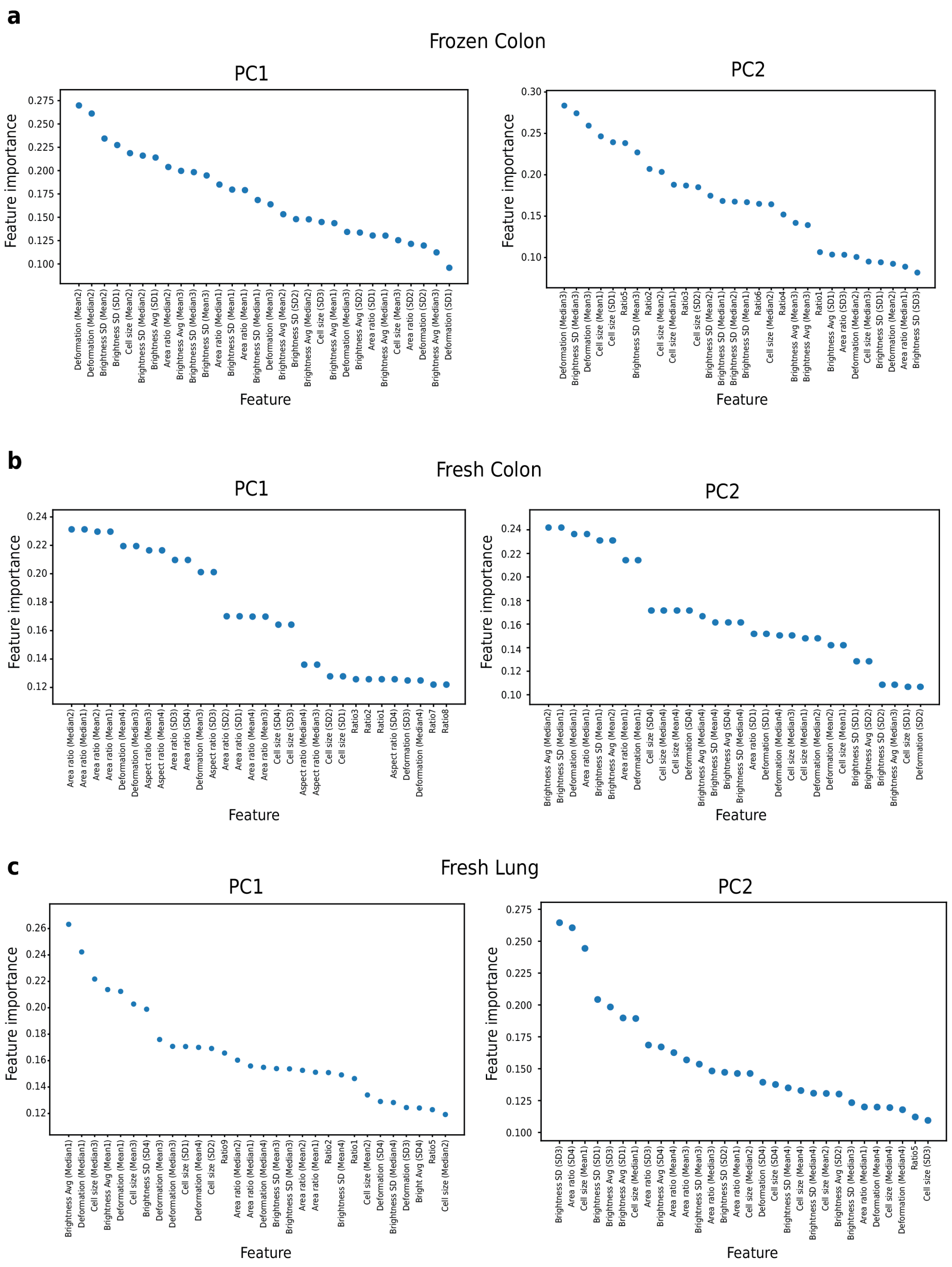

Supplementary Fig. 5 | Principal component feature importance for human biopsy samples. Relative feature importance for PC1 and PC2 for **a,** frozen colon biopsy samples, **b,** fresh colon biopsy samples, **c,** fresh lung biopsy samples. The number at the end of each feature label describes which bin of cell sizes it corresponds to (the bins are given in Fig. 5). Frozen colon: 1: 20-60; 2: 60-100; 3: 100-600 μm^2. Fresh colon: i.e. 1: 20-50; 2: 50-600 μm^2. Fresh Lung: 1: 20-60; 2: 60-100; 3: 100-200. 4: 200-600 μm^2. SD: standard deviation.

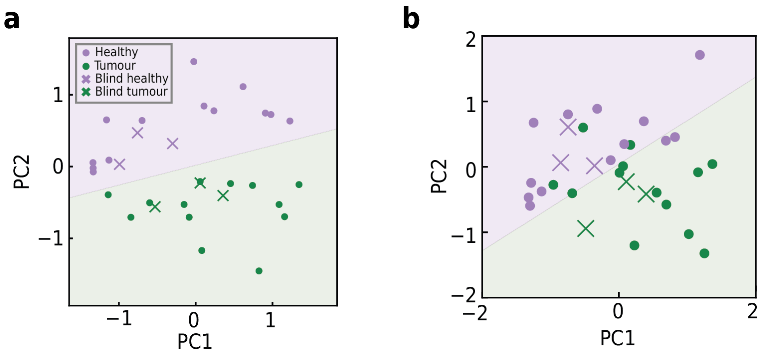

Supplementary Fig. 6 | Testing the performance of healthy vs tumour classification of frozen human colon samples. a, PCA and logistic regression including the full set of 45 parameters as input for PCA; **b,** PCA and logistic regression after excluding 3 parameters (deformation mean, median and SD for cells larger than 100 μm^2).

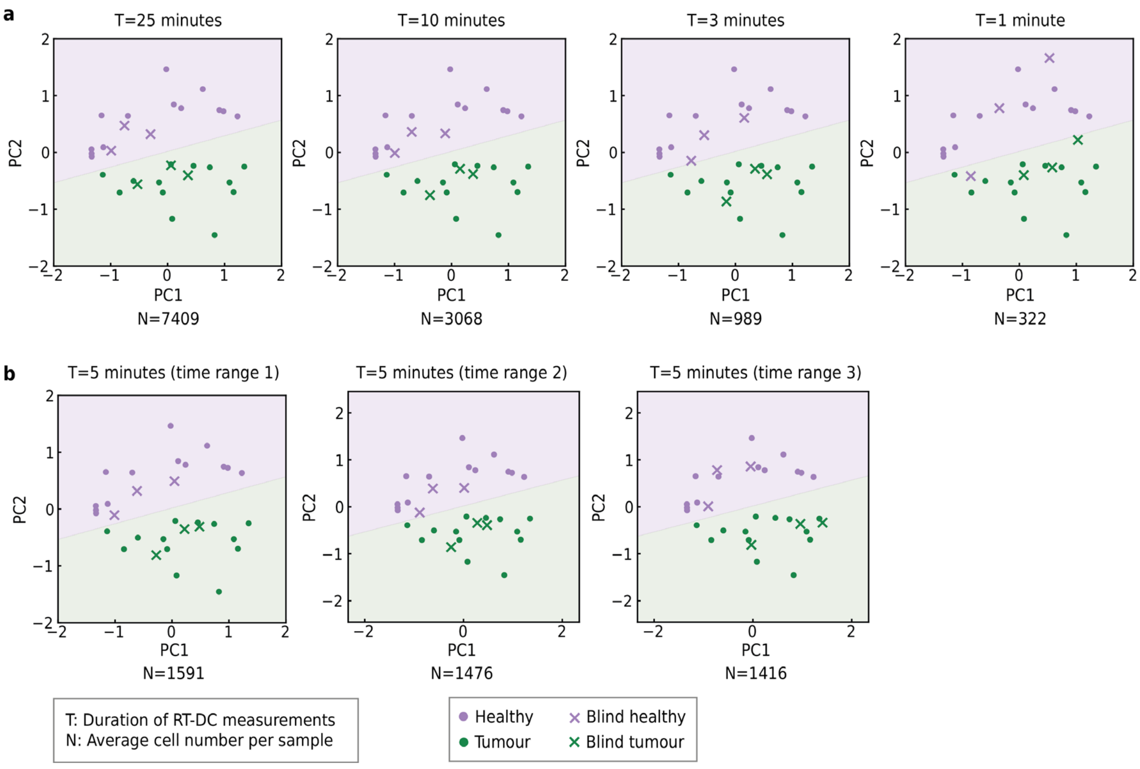

Supplementary Fig. 7 | Testing the performance of healthy vs tumour classification on reduced data from frozen human colon samples. a, Logistic regression was performed on the PCA (shown by the linear divide in the PCA plot) and used to predict the classification of six blind samples (shown as crosses). From left to right, the amount of data used for classification decreases (the plots correspond to experiment durations of T = 25, 10, 3 and 1 minute). The average cell numbers analysed for a sample are shown below each plot (N). Classification was correct for experiments lasting 3 minutes or longer; data yielded by a one-minute experiment was insufficient for 100% correct classification of healthy vs tumour samples. **b,** PCA plots performed using approximately 1500 cells (or an equivalent of 5 minutes of measurement) extracted from different time points of longer RT-FDC measurements.

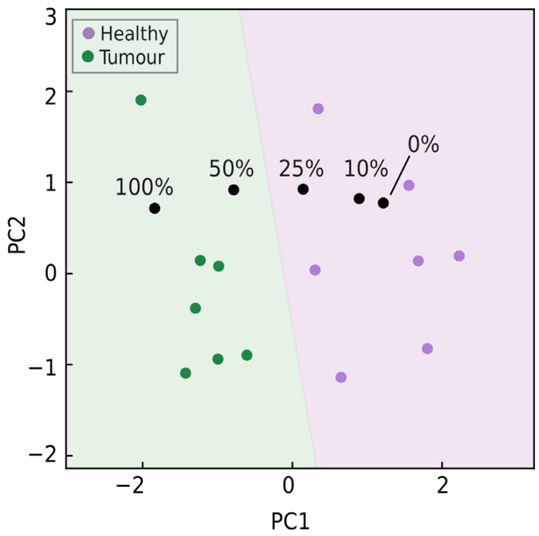

Supplementary Fig. 8 | Testing of the performance of logistic regression on mixed samples consisting of different ratios of tumour and healthy lung biopsy samples. The percentages indicate the percentage of the volume of the dissociated tumour sample in the total sample volume. The pure tumour sample and the sample with 50% tumour content were classified as tumour, while 25%, 10% and 0% of tumour content resulted in a classification in the “healthy” category.

Supplementary Tables

Supplementary Table 1 | Standard dissociation protocols used for each organ; *n* indicates the number of biological repeats per tissue.

Tissue	Dissociation procedure	*n*
**Colon**	Miltenyi Biotec Lamina Propria Dissociation Kit	4
**Small Intestine**	Miltenyi Biotec Lamina Propria Dissociation Kit	4
**Lung**	Miltenyi Biotec Lung Dissociation kit	4
**Liver**	Miltenyi Biotec Liver Dissociation Kit or Collagenase/Dispase enzymatic cocktail	4
**Stomach**	Ruiz et al 2012[1]	3
**Kidney**	Valente et al 2011[2]	3
**Pancreas**	Epshtein et al 2017[3]	3
**Thymus**	Mash tissue between frosted ends of two microscope slides	4
**Mesenteric Lymph Node**	Mash tissue between frosted ends of two microscope slides	4
**Spleen**	Mash tissue between frosted ends of two microscope slides	4

Supplementary Table 2 | Mouse organs with the corresponding tissue grinder protocols used for mechanical dissociation, together with the microfluidic chip size used for RT-FDC measurement; *n* indicates the number of biological repeats per tissue.

Tissue	Tissue grinder protocol	Microfluidic chip size	*n*
**Colon**	Colon	20 µm	4
**Small Intestine**	Intestine	20 µm	4
**Lung**	Lung standard	30 µm	4
**Liver**	Liver standard	30 µm & 40 µm	4
**Kidney**	Medium	30 µm	5
**Pancreas**	Soft	30 µm	4
**Thymus**	Thymus Soft	30 µm	4
**Mesenteric Lymph Node**	Lymph nodes standard	30 µm	4
**Spleen**	Thymus Soft	30 µm	5
**Stomach**	Intestine	30 µm	4
**Human tumour**	Human Tumour	20 µm	-

Supplementary Table 3 | Parameters derived from cell images in real-time during an RT-FDC measurement.

RT-FDC parameters	Description
**Area (cell size; μm^2)**	The projected cross-sectional area of the cells is obtained from the number of pixels within the boundary of the cell, defined by the contour
Bounding box size x* and *y	A bounding box around the whole cell defines its length in x and y direction
**Aspect ratio**	Ratio between object's length (x) and height (y)
**Circularity**	Parameter that quantifies the deviation from a circular cross-section, by relating area (*A*) to the perimeter (*P*) of an object based on $C = \frac{2\sqrt{\pi A}}{P}$
**Deformation**	$\frac{1 - 2\sqrt{\pi A}}{P}$
**Inertia ratio**	Ratio of the second moment of area over the y- and x-axis of the contour.
**Area ratio**	Ratio between the area of the convex hull and the area of the contour
**Brightness [a.u.]**	Mean of all pixel intensities inside the contour
**Standard deviation of brightness**	Standard deviation of the brightness of pixels inside the contour
**X position**	Position along the flow axis in the field of view.
**Y position**	Position perpendicular to flow axis in the field view
**Volume**	The apparent volume based on the assumption of rotational symmetry over the flow axis.
**Elastic modulus (Young's Modulus)**	Describes cell stiffness, based on assumptions of a model
**Fluorescence area of peak [μs]**	Area of the fluorescence intensity peak
**Fluorescence maximum [a.u.]**	Maximum fluorescence intensity
**Fluorescence width [μs]**	Full width at half-maximum; used to identify the sub-cellular distribution of fluorescence marker

Supplementary Table 4 | Antibodies and fluorescent probe solutions used with the corresponding dilutions.

	Reactivity	Dilution	Clone	Cat. No	Company
CD326 (EpCAM) Alexa Fluor® 488	Human	1 in 100	9C4	324210	BioLegend
CD31(PECAM-1) APC	Human	5µl/reaction	WM59	303115	BioLegend
CD45 PE	Human	1 in 500	HI30	304008	BioLegend
CD326 (EpCAM) FITC	Human	1 in 100	9C4	324203	BioLegend
CD45 Alexa Fluor® 700	Human	5µl/reaction	HI30	304024	BioLegend
CD45 Alexa Fluor® 700	Mouse	1 in 1000	30-F11	103128	BioLegend
CD326 (EpCAM) Alexa Fluor® 488	Mouse	1 in 200	G8.8	118210	BioLegend
CD45 FITC	Mouse	1 in 800	30-F11	11-0451-82	Thermo Fischer Scientific
CD326 (EpCAM) APC	Mouse	1 in 500	G8.8	17-5791-82	Thermo Fischer Scientific
CD31 (PECAM-1) PE	Mouse	1 in 250	390	12-0311-82	Thermo Fischer Scientific
DRAQ5™	-	5µl/reaction	-	65-0880-92	Thermo Fischer Scientific
Propidium Iodide	-	1 in 2000	-	P1304MP	Thermo Fischer Scientific
7-AAD Viability Solution	-	5µl/reaction	-	420404	Thermo Fischer Scientific

Supplementary Table 5 | Pathological information and stromal content evaluation of frozen human colon biopsy samples. sTILs: stromal tumour infiltrating lymphocytes (scored as percent of total tumour stroma occupied by tumour infiltrating lymphocytes). M: Male, F: Female. Samples were frozen prior to mechanical dissociation and analysis with RT-FDC.

Sample Number	Sex	Age	Diagnosis	Stromal Content (%)	Stroma Appearance	sTILs (%)	Localization of Biopsy
S1_Colon_Frozen	M	81	Colonic Adenocarcinoma	10	Fibrous	25	Colon Transversum
S3_Colon_Frozen	M	77	Colonic Adenocarcinoma	20	Fibrous	5	Colon Sigmoidoum
S4_Colon_Frozen	M	61	Colonic Adenocarcinoma	5	Fibrous	3	Colon Sigmoidoum
S5_Colon_Frozen	M	71	Colonic Adenocarcinoma	30	Fibrous	3	Colon Ascendens
S6_Colon_Frozen	M	84	Colonic Adenocarcinoma	40	Fibrous	40	Colon Ascendens
S7_Colon_Frozen	M	83	Rectal Adenocarcinoma	98	Fibrous	2	Rectum
S8_Colon_Frozen	F	57	Colonic Adenocarcinoma	30	Leiomyomatous	15	Rectum
S9_Colon_Frozen	M	69	Colonic Adenocarcinoma	35	Chondromyxoid	3	Colon Sigmoidoum
S11_Colon_Frozen	M	85	Rectal Adenocarcinoma	80	Chondromyxoid	3	Rectum
S12_Colon_Frozen	M	86	Colonic Adenocarcinoma	60	Leiomyomatous	40	Colon Transversum
S13_Colon_Frozen	M	73	Colonic Adenocarcinoma	25	Fibrous	30	Colon Ascendens
S14_Colon_Frozen	M	69	Colonic Adenocarcinoma	20	Leiomyomatous	15	Colon Transversum
S16_Colon_Frozen	M	88	Colonic Adenocarcinoma	15	Fibrous	5	Colon Ascendens

Supplementary Table 6 | Pathological information and stromal content evaluation of frozen human colon biopsy samples. sTILs: stromal tumour infiltrating lymphocytes (scored as percent of total tumour stroma occupied by tumour infiltrating lymphocytes). M: Male, F: Female. Samples were immediately processed with the tissue grinder and analysed with RT-FDC without prior freezing. Blue shaded samples were used as blind samples.

Sample Number	Sex	Age	Diagnosis	Stromal Content (%)	Stroma Appearance	sTILs (%)	Localization of Biopsy
S1_Colon_Fresh	F	62	Colonic Adenocarcinoma	40	Myxoid	15	Colon
S2_Colon_Fresh	M	76	Colonic Adenocarcinoma	30	Fibrous	15	Colon
S3_Colon_Fresh	M	81	Colonic Adenocarcinoma	10	Fibrous	25	Colon
S4_Colon_Fresh	M	85	Colonic Adenocarcinoma	25	Leiomyomatous	15	Colon
S5_Colon_Fresh	F	68	Colonic Adenocarcinoma	15	Fibrous	25	Sigma-Colon
S6_Colon_Fresh	M	83	Adenocarcinoma	10	Fibrous	5	Colon
S7_Colon_Fresh	M	60	Colonic Adenocarcinoma	25	Fibrous	35	Colon
S8_Colon_Fresh	M	61	Colonic Adenocarcinoma	45	Fibrous	25	Colon
S9_Colon_Fresh	F	61	Colonic Adenocarcinoma	15	Fibrous	10	Colon
S10_Colon_Fresh	F	69	Colonic Adenocarcinoma	10	Fibrous	5	Colon
S11_Colon_Fresh	F	79	Colonic Adenocarcinoma	25	Fibrous	7	Colon
S12_Colon_Fresh	F	81	Colonic Adenoma - high grade	20	Fibrous	60	Colon
S13_Colon_Fresh	F	69	Colonic Adenocarcinoma	5	Fibrous	5	Colon
S14_Colon_Fresh	F	70	Colonic Adenocarcinoma	5	Fibrous	25	Colon
S15_Colon_Fresh	F	83	Colonic Adenocarcinoma	5	Fibrous	50	Colon
S16_Colon_Fresh	M	77	Colonic Adenocarcinoma	10	Fibrous	15	Colon

Supplementary Table 7 | Histopathological evaluation of frozen human colon biopsy samples. T: tumour classification (ranges from T1 to T4). Grade 1: tumour cells look like healthy cells. Grade 2: tumour cells are somewhat abnormal. Grade 3: tumour cells and tissue look very abnormal. Grade 4: tumour cells are very abnormal. N: lymph node classification that assess infiltration of locoregional lymph nodes by the subsequent carcinoma/tumour (0=no lymph node metastases) The numbers in parentheses reflect the number of infiltrated lymph nodes (before the slash) and the number of evaluated lymph nodes (after the slash). L: Lymphovascular invasion; V: Blood Vessel invasion; Pn: perineural invasion; 0 means no invasion present, 1= invasion present; R= resection status; R0 = tumour is completely resected. NOS: not otherwise specified.

Sample Number	Histology	pT-Stage	pN-Stage	Grading	L	V	Pn	R
S1_Colon_Frozen	Intestinal Adenocarcinoma - Mucinous	pT2a	pN0 (0/21)	G2	0	0	0	0
S3_Colon_Frozen	Intestinal Adenocarcinoma - NOS	pT3c	pN0 (0/20)	G2	0	0	0	0
S4_Colon_Frozen	Intestinal Adenocarcinoma - NOS	pT3b	pN0 (0/33)	G2	0	0	0	0
S5_Colon_Frozen	Intestinal Adenocarcinoma - NOS	pT3b	pN0 (0/17)	G3	0	0	0	0
S6_Colon_Frozen	Intestinal Adenocarcinoma - Mucinous	pT3c	pN2b (10/12)	G3	1	1	0	0
S7_Colon_Frozen	Intestinal Adenocarcinoma - NOS	ypT2a	ypN1b (2/23)	G2	0	0	0	0
S8_Colon_Frozen	Intestinal Adenocarcinoma - NOS	pT2a	pN0 (0/36)	G2	0	0	0	0
S9_Colon_Frozen	Intestinal Adenocarcinoma - NOS	pT3b	pN2a (4/20)	G3	0	0	1	0
S11_Colon_Frozen	Intestinal Adenocarcinoma - NOS	ypT3b	pN0 (0/33)	G2	0	0	0	0
S12_Colon_Frozen	Intestinal Adenocarcinoma - NOS	pT2b	pN1a (1/24)	G3	1	1	0	0
S13_Colon_Frozen	Intestinal Adenocarcinoma - NOS	pT2b	pN0 (0/42)	G3	0	0	0	0
S14_Colon_Frozen	Intestinal Adenocarcinoma - NOS	pT4b	pN0 (0/18)	G2	1	0	0	0
S16_Colon_Frozen	Intestinal Adenocarcinoma - NOS	pT4a	pN2b (4/17)	G3	1	0	0	0

Supplementary Table 8 | Histopathological evaluation of fresh human colon biopsy samples. T: tumour classification (ranges from T1 to T4). Grade 1: tumour cells look like healthy cells. Grade 2: tumour cells are somewhat abnormal. Grade 3: tumour cells and tissue look very abnormal. Grade 4: tumour cells are very abnormal. N: lymph node classification that assess infiltration of locoregional lymph nodes by the subsequent carcinoma/tumour (0=no lymph node metastases) The numbers in parentheses reflect the number of infiltrated lymph nodes (before the slash) and the number of evaluated lymph nodes (after the slash). L: Lymphovascular invasion; V: Blood Vessel invasion; Pn: perineural invasion; 0 means no invasion present, 1= invasion present; R= resection status; R0 = tumour is completely resected. NOS: not otherwise specified. Blue shaded samples were used as blind samples.

Sample Number	Histology	pT-Stage	pN-Stage	Grading	L	V	Pn	R
S1_Colon_Fresh	Intestinal Adenocarcinoma - NOS	pT4a	pN2a (6/30)	G3	1	1	1	0
S2_Colon_Fresh	Intestinal Adenocarcinoma - NOS	pT3a	pN0 (0/29)	G2	0	0	0	0
S3_Colon_Fresh	Intestinal Adenocarcinoma - NOS	pT3b	pN1b (3/28)	G2	1	0	0	0
S4_Colon_Fresh	Intestinal Adenocarcinoma - NOS	pT3a	pN0 (0/12)	G2	0	0	0	0
S5_Colon_Fresh	Intestinal Adenocarcinoma - NOS	pT3a	pN2b (7/16)	G3	1	1	1	0
S6_Colon_Fresh	Intestinal Adenocarcinoma - NOS	pT3c	pN0 (0/16)	G2	0	0	0	0
S7_Colon_Fresh	Intestinal Adenocarcinoma - Mucinous	pT3c	pN1a (1/22)	G3	0	0	0	0
S8_Colon_Fresh	Intestinal Adenocarcinoma - NOS	pT4a	pN0 (0/27)	G3	0	0	0	0
S9_Colon_Fresh	Intestinal Adenocarcinoma - NOS	pT2	pN1a (1/18)	G3	0	0	0	0
S10_Colon_Fresh	Intestinal Adenocarcinoma - Mucinous	pT3c	pN0 (0/17)	G3	0	0	0	0
S11_Colon_Fresh	Intestinal Adenocarcinoma - NOS	pT3d	pN0 (0/33)	G2	0	0	0	0
S13_Colon_Fresh	Colonic Adenoma - high grade	N/A	N/A	high grade	N/A	N/A	N/A	N/A
S14_Colon_Fresh	Intestinal Adenocarcinoma - Mucinous/Gastric Type	pT4a	pN1a (1/33)	G3	0	0	1	0
S15_Colon_Fresh	Intestinal Adenocarcinoma - Medullary Type	pT3d	pN1a (1/32)	G3	1	0	0	0
S16_Colon_Fresh	Intestinal Adenocarcinoma - NOS	pT2b	pN0 (0/23)	G2	0	0	0	0

Supplementary Table 9 | Pathological information and stromal content evaluation of fresh human lung biopsy samples. sTILs: stromal tumour infiltrating lymphocytes (scored as percent of total tumour stroma occupied by tumour infiltrating lymphocytes). M: Male, F: Female. NSCLC: non-small cell lung cancer; SCLC: small cell lung cancer. Samples were immediately processed with the tissue grinder and analysed with RT-FDC without prior freezing. Blue shaded samples were used as blind samples.

Sample Number	Sex	Age	Diagnosis	Stromal Content (%)	Stroma Appearance	sTILs (%)	Localization of Biopsy
S1_Lung_Fresh	M	71	NSCLC - Adenocarcinoma	25	Fibrous	100	Pulmonary Lymph Node
S2_Lung_Fresh	M	85	NSCLC - Squamous Cell Carcinoma	15	Chondromyxoid	35	Lung
S4_Lung_Fresh	M	60	NSCLC - Adenosquamous Carcinoma	5	Fibrous	1	Lung
S5_Lung_Fresh	M	81	NSCLC - Squamous Cell Carcinoma	25	Chondromyxoid	15	Lung
S6_Lung_Fresh	M	64	NSCLC - Adenocarcinoma	50	Fibrous	50	Lung
S7_Lung_Fresh	M	72	NSCLC - Squamous Cell Carcinoma	10	Fibrous	1	Lung
S8_Lung_Fresh	F	76	SCLC	1	Fibrous	15	Lung
S9_Lung_Fresh	M	70	NSCLC - Squamous Cell Carcinoma	30	Fibrous	3	Lung

Supplementary Table 10 | Histopathological evaluation of fresh human lung biopsy samples. T: tumour classification (ranges from T1 to T4). Grade 1: tumour cells look like healthy cells. Grade 2: tumour cells are somewhat abnormal. Grade 3: tumour cells and tissue look very abnormal. Grade 4: tumour cells are very abnormal. N: lymph node classification that assess infiltration of locoregional lymph nodes by the subsequent carcinoma/tumour (0=no lymph node metastases) The numbers in parentheses reflect the number of infiltrated lymph nodes (before the slash) and the number of evaluated lymph nodes (after the slash). L: Lymphovascular invasion; V: Blood Vessel invasion; Pn: perineural invasion; 0 means no invasion present, 1= invasion present; R= resection status; R0 = tumour is completely resected. Blue shaded samples were used as blind samples.

Sample Number	Histology	pT-Stage	pN-Stage	Grading	L	V	Pn	R
S1_Lung_Fresh	Primary Pulmonary Adenocarcinoma - Solid Type	TX	pN2 (2/10)	G3	1	0	0	0
S2_Lung_Fresh	Primary Pulmonary Squamous Cell Carcinoma	pT3	pN0 (0/56)	G2	1	0	0	0
S4_Lung_Fresh	Primary Pulmonary Adenosquamous Carcinoma	pT4	pN1 (1/10)	G3	1	0	1	0
S5_Lung_Fresh	Primary Pulmonary Squamous Cell Carcinoma	pT2b	pN0 (0/34)	G3	0	0	0	0
S6_Lung_Fresh	Primary Pulmonary Adenocarcinoma - Lepidic Type	pT1c	pN0 (0/29)	G1	0	0	1	0
S7_Lung_Fresh	Primary Pulmonary Squamous Cell Carcinoma	pT4	pN0 (0/18)	G3	0	0	0	0
S8_Lung_Fresh	Small Cell Neuroendocrine Carcinoma	pT1c	pN0 (0/18)	G3	0	0	0	0
S9_Lung_Fresh	NSCLC - Squamous Cell Carcinoma	pT1c	N/A	G3	0	0	0	0

Supplementary Table 11 | Deformability Cytometry Open Repository (DCOR) dataset identifiers[4].

Dataset name	DCOR Identifier
Figure 2 - Liver, colon, kidney data	https://dcor.mpl.mpg.de/defc199e-d465-4254-9b65-973408f7843a
Figure 3 - Control group (murine colon samples)	https://dcor.mpl.mpg.de/d2eb46c2-92d4-41a8-aed0-1b77c6a43ba6
Figure 3 - Transfer colitis (murine colon samples)	https://dcor.mpl.mpg.de/04c95441-311e-47bf-b122-01d9e6c5d1f4
Figure 4 - Control group (murine colon samples)	https://dcor.mpl.mpg.de/35af55de-9f5c-4a57-8b9b-55eefade4613
Figure 4 - Tumour (murine colon samples)	https://dcor.mpl.mpg.de/0f1544ae-00d4-4e2b-b413-737aa034eb3a
Figure 5 - Frozen human colon samples – healthy	https://dcor.mpl.mpg.de/b3512170-77d8-4c8e-8e52-b407f841e57e
Figure 5 - Frozen human colon samples – tumour	https://dcor.mpl.mpg.de/578beb51-885b-443f-b847-94605de78390
Figure 5 - Fresh human colon samples – healthy	https://dcor.mpl.mpg.de/9a6439a2-0220-40cc-a61e-dd0407aeedc9
Figure 5 - Fresh human colon samples – tumour	https://dcor.mpl.mpg.de/84a6f22c-1df4-4add-b3b5-a4efe3c0adb1
Figure 5 - Fresh human lung samples – healthy	https://dcor.mpl.mpg.de/d8d247d7-b2f4-406c-b7c2-43770653a16d
Figure 5 - Fresh human lung samples – tumour	https://dcor.mpl.mpg.de/fb460cd1-e82e-4963-bc51-9bf83d3ea013
Figure 5 – Frozen human colon - blind samples	https://dcor.mpl.mpg.de/342b1d2e-5e1d-47ed-88c2-7871243f739a
Figure 5 - Fresh human colon - blind samples	https://dcor.mpl.mpg.de/498112f0-b7b9-45cc-b71f-79e729bbfc30
Figure 5 - Fresh human lung - blind samples	https://dcor.mpl.mpg.de/a3698039-e441-43fc-aa4d-67833d0ae942
Extended Data Figure 3 - Cell doublets	https://dcor.mpl.mpg.de/96ade6cb-3d46-40fd-88b6-d9c59a9ae330
Extended Data Figure 4 - Liver and lung	https://dcor.mpl.mpg.de/03c49f24-8d41-445b-aae3-a18790bfe82e
Extended Data Figure 5 - Comparison of fresh and frozen human colon	https://dcor.mpl.mpg.de/5cc528df-732f-4035-a99f-7f004ae3e37c

References

1. Ruiz, V. E., Sachdev, M., Zhang, S., Wen, S. & Moss, S. F. Isolating, immunophenotyping and ex vivo stimulation of CD4+ and CD8+ gastric lymphocytes during murine Helicobacter pylori infection. *J. Immunol. Methods* **384**, 157–163 (2012).

2. Valente, M. J. *et al.* A Rapid and Simple Procedure for the Establishment of Human Normal and Cancer Renal Primary Cell Cultures from Surgical Specimens. *PLoS One* **6**, e19337 (2011).

3. Epshtein, A., Sakhneny, L. & Landsman, L. Isolating and Analyzing Cells of the Pancreas Mesenchyme by Flow Cytometry. *J. Vis. Exp.* 5–11 (2017) doi:10.3791/55344.

4. Soteriou, D. *et al.* Datasets for: Rapid single-cell physical phenotyping of mechanically dissociated tissue biopsies. *Deformability Cytometry Open Repository (DCOR)* https://dcor.mpl.mpg.de/organization/soteriou-kubankova (2023).
