# Supplementary material for: Rapid single-cell physical phenotyping of mechanically dissociated tissue biopsies
Source: Nat Biomed Eng. 2023 Apr 6;7(11):1392–403. doi: 10.1038/s41551-023-01015-3 (PMC10651479; doi:10.1038/s41551-023-01015-3)
Supplement: Supplementary file 2 — Reporting Summary [file 41551_2023_1015_MOESM2_ESM.pdf]

nature portfolio

Corresponding author(s): Jochen Guck

Last updated by author(s): Jan 24, 2023

Reporting Summary

Nature Portfolio wishes to improve the reproducibility of the work that we publish. This form provides structure for consistency and transparency in reporting. For further information on Nature Portfolio policies, see our Editorial Policies and the Editorial Policy Checklist.

Statistics

For all statistical analyses, confirm that the following items are present in the figure legend, table legend, main text, or Methods section.

n/a	Confirmed	
☐	☒	The exact sample size (n) for each experimental group/condition, given as a discrete number and unit of measurement
☒	☐	A statement on whether measurements were taken from distinct samples or whether the same sample was measured repeatedly
☐	☒	The statistical test(s) used AND whether they are one- or two-sided<br>*Only common tests should be described solely by name; describe more complex techniques in the Methods section.*
☒	☐	A description of all covariates tested
☒	☐	A description of any assumptions or corrections, such as tests of normality and adjustment for multiple comparisons
☐	☒	A full description of the statistical parameters including central tendency (e.g. means) or other basic estimates (e.g. regression coefficient) AND variation (e.g. standard deviation) or associated estimates of uncertainty (e.g. confidence intervals)
☐	☒	For null hypothesis testing, the test statistic (e.g. F, t, r) with confidence intervals, effect sizes, degrees of freedom and P value noted<br>*Give P values as exact values whenever suitable.*
☒	☐	For Bayesian analysis, information on the choice of priors and Markov chain Monte Carlo settings
☒	☐	For hierarchical and complex designs, identification of the appropriate level for tests and full reporting of outcomes
☐	☒	Estimates of effect sizes (e.g. Cohen's d, Pearson's r), indicating how they were calculated

Our web collection on statistics for biologists contains articles on many of the points above.

Software and code

Policy information about availability of computer code

Data collection	We used ShapeIn (ShapeIn2; commercially available from Zellmechanik Dresden GmbH) for the acquisition of the RT-FDC data.
Data analysis	Python 3.7 was used. The Python library dclab is open-source and available on github (https://github.com/ZELLMECHANIK-DRESDEN/dclab); package version 0.32.3 was used for data analysis. We performed statistical analyses with the SciPy 1.3.0 package. Additional data analysis was performed with Scikit learn 0.23.2 package (https://scikit-learn.org/stable/).<br>The Python code for the processing and visualisation of RT-FDC data is available at https://github.com/marketakub/physical_phenotyping_tissues.

For manuscripts utilizing custom algorithms or software that are central to the research but not yet described in published literature, software must be made available to editors and reviewers. We strongly encourage code deposition in a community repository (e.g. GitHub). See the Nature Portfolio guidelines for submitting code & software for further information.

Data

Policy information about availability of data

All manuscripts must include a data availability statement. This statement should provide the following information, where applicable:

- Accession codes, unique identifiers, or web links for publicly available datasets
- A description of any restrictions on data availability
- For clinical datasets or third party data, please ensure that the statement adheres to our policy

The RT-FDC datasets generated and analysed for Figs. 2–5 and Extended Data Figs. 3–5 are available on the Deformability Cytometry Open Repository (https://dcor.mpl.mpg.de/organization/soteriou-kubankova)73. Individual identifiers for each dataset are provided in Supplementary Table 11. Source data for Extended Data Fig. 1 are also provided with this paper.

Human research participants

Policy information about studies involving human research participants and Sex and Gender in Research.

Reporting on sex and gender	Sex and gender were not considered in the study design nor in the data analysis. Our working assumption was that there are no sex-related or gender-related differences in colon tumours; hence, sex and gender were not considered as relevant parameters in our analysis.
Population characteristics	Surgically resected human biopsy samples from male and female patients of age range 57–88 were obtained from the Pathology Institute, Erlangen. All samples used were obtained from leftover biopsy samples. Therefore, this work did not interfere with standard practices of care or with sample-collection procedures. We provide the main population characteristics, including age, gender, diagnosis, localization of biopsy, histology, pT and pN stage, tumour grade, resection status, and the characteristics of the stroma and invasion state, in the Supplementary Information.
Recruitment	No active recruitment was needed. We used leftover biopsy samples that were obtained (subject to availability) from the Pathology Institute, Erlangen, following patient surgery. The biopsy samples were not collected specifically for this research study but were part of the standard practices of patient care. Informed consent was obtained from the patients providing samples. The participants were not compensated. All experiments were carried out in accordance with the declaration of Helsinki.
Ethics oversight	The study is covered by ethic votes of the University Hospital of the Friedrich-Alexander University Erlangen-Nurnberg (24.01.2005, 18.01.2012). The Institutional Review Board of the University Hospital of the Friedrich-Alexander University Erlangen-Nürnberg approved the study (ID: Re.-No. 4607).

Note that full information on the approval of the study protocol must also be provided in the manuscript.

Field-specific reporting

Please select the one below that is the best fit for your research. If you are not sure, read the appropriate sections before making your selection.

☒ Life sciences ☐ Behavioural & social sciences ☐ Ecological, evolutionary & environmental sciences

For a reference copy of the document with all sections, see nature.com/documents/nr-reporting-summary-flat.pdf

Life sciences study design

All studies must disclose on these points even when the disclosure is negative.

Sample size	No statistical method was used to predetermine sample sizes. The sample sizes used in the study are in accordance with past experience and with standard sizes used in real-time deformability-cytometry measurements, which are of the order of several hundreds of cells per measurement per condition. The number of mice used in the animal studies were estimated according to the Resource Equation method [Charan et al. (2013), J Pharmacol Pharmacother]. For human biopsies, sample sizes were determined by the number of patient samples available.
Data exclusions	For the analysis of real-time deformability-cytometry data, we excluded debris by filtering out events smaller than 20 µm^2 in cross-sectional area, a procedure commonly used in real-time deformability cytometry. An additional area-ratio filter of 1.0–1.1 was used to ensure that only events with correctly fitted contours are used in the data analysis, as previously established by ZellMechanik Dresden GMBH, Dresden, Germany.
Replication	All of the experiments presented in the manuscript were repeated more than once, as indicated in the figure legend. All experiments were included in the analysis.
Randomization	Randomization was not relevant for the study, because no treatment group was involved.

Blinding	For blind data analysis, samples were numerically tagged. The investigator running the analysis was unaware of which sample corresponded to tumour or healthy tissue. Only the investigators acquiring the data were aware of the status of the biopsy. Owing to the appearance of the biopsy samples, it was in some cases clear to the investigator performing the experiments which sample corresponded to tumour or healthy tissue. It was therefore impossible to perform blind data acquisition.

Reporting for specific materials, systems and methods

We require information from authors about some types of materials, experimental systems and methods used in many studies. Here, indicate whether each material, system or method listed is relevant to your study. If you are not sure if a list item applies to your research, read the appropriate section before selecting a response.

Materials & experimental systems

n/a	Involved in the study
☐	☒ Antibodies
☒	☐ Eukaryotic cell lines
☒	☐ Palaeontology and archaeology
☐	☒ Animals and other organisms
☒	☐ Clinical data
☒	☐ Dual use research of concern

Methods

n/a	Involved in the study
☒	☐ ChIP-seq
☒	☐ Flow cytometry
☒	☐ MRI-based neuroimaging

Antibodies

Antibodies used	anti-human: CD326 (EpCAM) Alexa Fluor 488 (1:100, Clone:9C4, Ref :324210; BioLegend, CA, USA); CD31(PECAM-1) APC (5µl/reaction, Clone: WM59, Ref: 303115; BioLegend, CA, USA); CD45-PE (1:500. Clone HI30, Ref: 304008; BioLegend, CA, USA); CD326 (EpCAM) FITC (1:500, Clone:9C4, Ref: 324203; BioLegend, CA, USA); CD45 Alexa Fluor 700 (5µl/reaction, Clone: HI30, Ref: 304024; BioLegend, CA, USA). Anti-mouse: CD45 Alexa Fluor® 700 (1:1000, Clone: 30-F11, Ref: 103128; BioLegend, CA, USA); CD326 (EpCAM) Alexa Fluor 488 (1:200, Clone: G8.8, Ref: 118210; BioLegend, CA, USA); CD45-FITC (1:800. Clone: 30-F11, Ref: 11-0451-82; Thermo Fischer Scientific, MA, USA); CD326 (EpCAM) APC (1:500, Clone: G8.8, Ref: 17-5791-82; Thermo Fischer Scientific, MA, USA); CD31 (PECAM) PE (1:250, Clone:390, Ref: 12-0311-82; Thermo Fischer Scientific, MA, USA).
Validation	Antibodies were validated by the manufacturers and were used according to manufacturers' recommended dilutions.<br>Anti-human:<br>CD326 (EpCAM) Alexa Fluor 488: https://www.biolegend.com/en-ie/search-results/alexa-fluor-488-anti-human-cd326-epcam-antibody-3759<br>CD31(PECAM-1) APC: https://www.biolegend.com/en-ie/products/apc-anti-human-cd31-antibody-6123<br>CD45 PE: https://www.biolegend.com/en-ie/products/pe-anti-human-cd45-antibody-708<br>CD326 (EpCAM) FITC: https://www.biolegend.com/en-ie/products/fitc-anti-human-cd326-epcam-antibody-3756<br>CD45 Alexa Fluor 700: https://www.biolegend.com/en-ie/products/alexa-fluor-700-anti-human-cd45-antibody-3401<br><br>Anti-mouse:<br>CD45 Alexa Fluor 700 : https://www.biolegend.com/en-ie/products/alexa-fluor-700-anti-mouse-cd45-antibody-3407<br>CD326 (EpCAM) Alexa Fluor 488 :https://www.biolegend.com/en-ie/products/alexa-fluor-488-anti-mouse-cd326-ep-cam-antibody-4972<br>CD45 FITC: https://www.thermofisher.com/antibody/product/CD45-Antibody-clone-30-F11-Monoclonal/11-0451-82<br>CD326 (EpCAM) APC: https://www.thermofisher.com/antibody/product/CD326-EpCAM-Antibody-clone-G8-8-Monoclonal/17-5791-82<br>CD31 (PECAM-1) PE: https://www.thermofisher.com/antibody/product/CD31-PECAM-1-Antibody-clone-390-Monoclonal/12-0311-82

Animals and other research organisms

Policy information about studies involving animals; ARRIVE guidelines recommended for reporting animal research, and Sex and Gender in Research

Laboratory animals	For the comparison of enzymatic and tissue grinding: C57BL/6J females and males, age 8–19 weeks;<br>For the transfer colitis model: Mus musculus, Rag1-/-, female, 8–12 weeks at the start of the experiment.<br>For the tumour model: Mus musculus, female and male mice, 30–40 weeks.<br>Animals were housed in 12-hour light–dark cycle, at 20–23°C and 40–60 % humidity.
Wild animals	The study did not involve wild animals.
Reporting on sex	Male (N=3) and female (N=4) animals were used for the comparison of enzymatic dissociation and tissue grinding. For each tissue extracted, half were used for enzymatic dissociation and half for tissue grinding.<br><br>Only female animals were used for the transfer colitis experiments (N=14), as described in the original publication (DOI: 10.1093/intimm/5.11.1461)

	Both male (N=7) and female (N=9) animals were used for the tumour model.<br><br>No sex-based analysis was performed because this was beyond the scope of this study.
Field-collected samples	The study did not involve samples collected from the field.
Ethics oversight	The experiments were performed in accordance to the guidelines of the Institutional Animal Care and Use Committee of the State Government of Middle Franconia.<br>Transfer colitis: TVA: 55.2.2- 2532-2-473; Government of Lower Franconia<br>Tumour model: TVA: 55.2.2- 2532-2-1032; Government of Lower Franconia

Note that full information on the approval of the study protocol must also be provided in the manuscript.
